# Oncogenic K‐Ras^G12V^ cannot overcome proliferation failure caused by loss of Ppp6c in mouse embryonic fibroblasts

**DOI:** 10.1002/2211-5463.13775

**Published:** 2024-02-06

**Authors:** Mai Ito, Nobuhiro Tanuma, Yui Kotani, Kokoro Murai, Ayumi Kondo, Mami Sumiyoshi, Hiroshi Shima, Satoshi Matsuda, Toshio Watanabe

**Affiliations:** ^1^ Department of Biological Science, Graduate School of Humanities and Sciences Nara Women's University Japan; ^2^ Division of Cancer Chemotherapy Miyagi Cancer Center Research Institute Natori Japan; ^3^ Department of Cell Signaling, Institute of Biomedical Science Kansai Medical University Hirakata Japan

**Keywords:** cell proliferation, K‐Ras^G12V^, MEF, PP6, Ppp6c, tumor formation

## Abstract

Protein phosphatase 6 is a Ser/Thr protein phosphatase and its catalytic subunit is Ppp6c. Ppp6c is thought to be indispensable for proper growth of normal cells. On the other hand, loss of Ppp6c accelerates growth of oncogenic Ras‐expressing cells. Although it has been studied in multiple contexts, the role(s) of Ppp6c in cell proliferation remains controversial. It is unclear how oncogenic K‐Ras overcomes cell proliferation failure induced by Ppp6c deficiency; therefore, in this study, we attempted to shed light on how oncogenic K‐Ras modulates tumor cell growth. Contrary to our expectations, loss of Ppp6c decreased proliferation, anchorage‐independent growth in soft agar, and tumor formation of oncogenic Ras‐expressing mouse embryonic fibroblasts (MEFs). These findings show that oncogenic K‐Ras^G12V^ cannot overcome proliferation failure caused by loss of Ppp6c in MEFs.

## Graphical abstract

Protein phosphatase 6 is a Ser/Thr protein phosphatase and its catalytic subunit is Ppp6c. Ppp6c is thought to be indispensable for proper growth of normal cells. On the other hand, loss of Ppp6c accelerates growth of oncogenic Ras‐expressing cells. Here we showed that oncogenic K‐Ras^G12V^ cannot overcome proliferation failure caused by loss of Ppp6c in mouse embryonic fibroblasts.

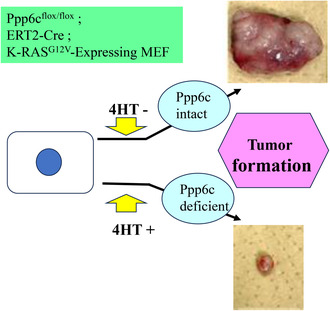

**Abbreviations**
4HT: 4‐hydroxytamoxifen
DepMap: Cancer Dependency Map
DMEM: Dulbecco's modified Eagle's medium
FCS: fetal calf serum
mAb: monoclonal antibody
MAPK: mitogen‐activated protein kinase
MEF: mouse embryonic fibroblast
PDAC: pancreatic invasive ductal adenocarcinoma
PP6: protein phosphatase 6

Protein phosphatase 6 (PP6) is a Ser/Thr protein phosphatase composed of three subunits: the catalytic subunit Ppp6c and the regulatory subunits Ankrds and SAPS [1]. The diverse phenotypes observed following small interfering RNA‐based Ppp6c knockdown in cultured mammalian cells suggest that PP6 regulates mitosis by dephosphorylating Aurora kinase A [2], activates DNA‐PK to sensitize cells to ionizing radiation [3], and is required for homology‐directed repair [4]. There is also evidence that PP6 regulates nuclear factor‐κB signaling by blocking IκBε degradation in response to tumor necrosis factor [5] and inactivating TAK1 [6]. Studies in flies indicate that depletion of PpV, the *Drosophila* homolog of Ppp6c, results in a cell growth defect [7], suggesting that Ppp6c promotes cell growth. We reported that Ppp6c is indispensable for proper post‐implantation mouse embryogenesis. Ppp6c deficiency greatly reduces proliferation of primary mouse embryonic fibroblasts (MEFs) and induces significant growth failure of the inner cell mass of blastocysts. [8]. Thus, Ppp6c is thought to be indispensable for proper growth of normal mouse cells.

Apart from the roles of PP6 in regulating the cell cycle and mitosis [2, 9], little is understood about how Ppp6c modulates tumor growth. We assessed Ppp6c function in a mouse model of skin carcinogenesis and found that loss of Ppp6c in keratinocytes promotes 7,12‐dimethylbenz[a]anthracene‐induced papilloma formation and ultraviolet B‐induced carcinogenesis [10, 11]. These findings support the idea that Ppp6c acts as a tumor suppressor in mouse skin cancers.

A recent large‐scale ethyl methanesulfonate‐induced genetic screen in Drosophila revealed that loss of PP6 cooperates with oncogenic Ras (RasV^12^) to induce tumor cell proliferation and invasion, suggesting that PP6 serves as a tumor suppressor in Ras‐related cancers [12]. We also reported that Ppp6c deficiency accelerates K‐Ras^G12D^‐induced keratinocyte tumor promotion and tongue carcinogenesis [13, 14]. These reports suggest that Ppp6c suppresses growth of oncogenic Ras‐expressing cells and loss of Ppp6c accelerates growth of these cells. Thus, Ppp6c is thought to have opposite effects on proliferation of normal and oncogenic K‐Ras‐expressing cells *in vitro* and *in vivo*.

Despite previous studies in many different contexts, the role(s) of Ppp6c in cell proliferation remains controversial. It is unclear how oncogenic K‐Ras expression overcomes cell proliferation failure induced by Ppp6c deficiency; therefore, we sought to shed light on how oncogenic K‐Ras modulates tumor cell growth. Here, we established CreERT‐Ppp6c^fl/fl^‐K‐Ras^G12V^ MEFs in which Ppp6c deletion was induced by addition of 4‐hydroxytamoxifen (4HT) and analyzed the effects of Ppp6c deficiency on growth of K‐Ras^G12V^‐expressing MEFs *in vitro* and *in vivo*. To our surprise, loss of Ppp6c dramatically decreased cell proliferation, anchorage‐independent growth in soft agar, and tumor formation in mice. Collectively, these findings show that oncogenic K‐Ras^G12V^ cannot overcome proliferation failure caused by loss of Ppp6c in MEFs.

## Materials and methods

### Ethics statement

This study was approved by the Committee of Animal Experiments, Nara Women's University (approval ID 18‐01).

### Retrovirus infection

CreERT‐Ppp6c^fl/fl^‐K‐Ras^G12V^ MEFs were produced by infecting a K‐Ras^G12V^‐expressing retrovirus into established CreERT‐Ppp6c^fl/fl^ MEFs [15]. Briefly, primary CreERT‐Ppp6c^fl/fl^ MEFs were generated from CreERT‐Ppp6c^fl/fl^ embryos at 14.5 days after conception. Primary CreERT‐Ppp6c^fl/fl^ MEFs were immortalized by transfection of a plasmid containing SV40 genomic DNA [16]. To prepare the retrovirus stock, PLAT‐E cells seeded into a 6 cm dish without blasticidin and puromycin were treated with 200 μL of serum‐free Dulbecco's modified Eagle's medium (DMEM) containing 3 μg of pBABE‐puro‐KrasV12 (https://www.addgene.org/9052/) and 20 μL of Fugene HD. After incubation for 24 h, the medium was changed. After an additional 24 h, the virus‐containing supernatant was collected. MEFs were infected in a 3.5 cm dish with 750 μL of retrovirus solution containing 8 μg·mL^−1^ polybrene (Sigma‐Aldrich, St. Louis, MO, USA). After incubation for 5 h, the medium was changed to fresh medium containing 2 μg·mL^−1^ puromycin.

### Induction of Ppp6c deletion in MEFs

4HT (Toronto Research Chemicals) was used to induce CreERT2‐dependent recombination, as reported previously [15]. MEFs were cultured in DMEM supplemented with 10% fetal calf serum (FCS) and antibiotics (penicillin/streptomycin) at 37 °C in 5% CO_2_. On Day 0, MEFs were seeded in a 6 cm dish such that they would be confluent on Day 4. On Day 1, the medium was changed to fresh medium containing or lacking 1 μm 4HT. On Day 2, the medium was changed to fresh medium lacking 4HT and the incubation was continued. On Day 4, MEFs were used for further analysis.

### PCR genotype analysis

To confirm deletion of exon 4 from floxed *Ppp6c*, the following primers were used: Ppp6c FW, AGTTCCTCAAGCTTACAGAGAAATAAC, and Ppp6c RV, AGGAACAGCATTCAGCCTTTC. To confirm pBABE‐*K‐RAS* ^*G12‐V*^, the following primers were used: K‐Ras FW, GTGGACGAATATGATCCAACA, and pBABE3, ACCCTAACTGACACACATTCC.

### Real‐time PCR

Total RNA was extracted from MEFs using ISOGEN II (Nippon Gene, Tokyo, Japan) and then reverse‐transcribed using ReverTra Ace‐α (Toyobo, Tokyo, Japan) according to the manufacturers' instructions. RT‐qPCR with SYBR Green detection was performed using a StepOne Real‐Time PCR System (Thermo Fisher, Waltham, MA, USA). Gene expression was normalized to that of *Gapdh*. The following primers were used: Ppp6cL, ACACAGGTGTATGGATTTTATGATG; Ppp6cR, TGAGCATATCAAAAACTTTGGTACAG; K‐RasG12VFW, CTTGTGGTAGTTGGAGCTGT; K‐RasG12VRV, GGAATCCTCTATTGTTGGA; GapdhFW, CACCATCTTCCAGGAGCGAG; and GapdhRV, CCTTCTCCATGGTGGTGAAGAC.

### Cell proliferation assay

MEFs were cultured in DMEM supplemented with 10% FCS and antibiotics (penicillin/streptomycin) at 37 °C in 5% CO_2_. Five hundred cells were seeded per well into a 96‐well plate and cultured for up to 5 days. Cell numbers were determined using a Cell Counting Kit‐8 (Dojindo Laboratories, Kumamoto, Japan) according to the manufacturer's instructions.

### Anchorage‐independent growth in 3D culture

One milliliter of 0.5% agarose‐S (Nippon Gene) in DMEM was solidified in each well of a 6‐well plate or 3.5 cm dishes (Grainer Bio‐One, Nürtingen, Germany) as the bottom agarose layer and incubated for 1 h at room temperature. The top agarose (0.4%) layer containing cells was poured over the bottom agarose layer (10 000 cells per well) and set for 30 min at room temperature. One milliliter of DMEM containing 10% FCS was overlayed. The samples were cultured in a humidified incubator at 37 °C with 5% CO_2_ for 20 days and the overlayed medium was removed. Colonies were stained for 20 min with phosphate‐buffered saline containing 0.5% Crystal Violet. Digital images of the colonies were acquired. Colonies were manually counted.

### Tumor formation of CreERT‐Ppp6c^fl^ ^/^ ^fl^‐K‐Ras^G12V^ MEFs in C57BL/6 mice

MEFs were processed to induce Ppp6c deletion and 2 × 10^6^ cells were suspended in 100 μL of serum‐free DMEM. Female C57BL/6 mice at 6–7 weeks of age were subcutaneously inoculated with MEFs (2 × 10^6^). After confirmation of visible tumors, tumor volumes were measured every 2 days using digital calipers. The tumor volume (mm^3^) was calculated using the following formula: 0.5 × (length) × (width)^2^. The final weight of the tumor was recorded.

### Preparation of cell lysates, SDS/PAGE, and western blotting

Cell lysates were prepared and immunoblot analysis was performed. Briefly, cells were lysed in RIPA Lysis Buffer (Santa Cruz Biotechnology, Nürtingen, Germany, USA) containing PMSF, a protease inhibitor cocktail, sodium orthovanadate, and a phosphatase inhibitor cocktail (Nacalai Tesque, Kyoto, Japan). The samples were centrifuged. The supernatants were collected, supplemented with 4× SDS sample buffer, and incubated at 95 °C. The samples were separated by SDS/PAGE and transferred to PVDF membranes (Bio‐Rad Laboratories, Hercules, CA, USA). The membranes were incubated in PVDF Blocking Reagent for Can Get Signal^®^ (TOYOBO) and then with a primary antibody for 1 h at room temperature. Membranes were subsequently incubated with the appropriate secondary antibody in Can Get Signal^®^ Solution 2 (TOYOBO) for 1 h at room temperature. Then, immunoreactive proteins were visualized by ImmunoStar^®^ LD (WAKO, Osaka, Japan) and detected with a Luminescent Image Analyzer System LAS‐3000 (FUJIFILM, Tokyo, Japan). For re‐probing, membranes were washed in 1× TBS‐T (Tris‐buffered saline containing 0.1% Tween^®^ 20) for 5 min (three times) and shaken in stripping solution [10 mL of 2% SDS containing 93 mm Tris–HCl (pH 6.8) and 0.7% 2‐mercaptoethanol] at 50 °C for 30 min. The membrane was then washed with 1× TBS‐T for 10 min (four times) and re‐detected to confirm that stripping was complete, and the blocking procedure was repeated.

### Antibodies

A polyclonal anti‐Ppp6c antibody against a peptide corresponding to the C‐terminal 16 amino acids was generated in our laboratory [10]. Other antibodies were purchased as follows: anti‐p44/42 mitogen‐activated protein kinase (MAPK) (237F5) rabbit monoclonal antibody (mAb) (#4695, CST, Danvers, MA, USA), anti‐SAPK/JNK antibody (#9252, CST), anti‐p38 MAPK (D13E1) XP rabbit mAb (#8690, CST), anti‐Akt antibody (#9272, CST), anti‐phospho‐p44/42 MAPK (Thr202/Tyr204) (D13, 14, 4E) XP rabbit mAb (#4370, CST), anti‐phospho‐p38 MAPK (Thr180/Tyr182) (D3F9) rabbit mAb (#4695, CST), anti‐phospho‐SAPK/JNK (Thr183/Thr185) (81E11) rabbit mAb (#4695, CST), anti‐phospho‐Akt (Ser473) (D9E) XP^®^ Rabbit mAb (#4060, CST), anti‐Ras (27H5) rabbit mAb (#3339S, CST), and anti‐β‐actin mouse mAb (AC‐15) (#A5441, Sigma‐Aldrich). Horseradish peroxidase‐conjugated anti‐rabbit IgG (#7074) and horseradish peroxidase‐conjugated anti‐mouse IgG (#NA931) were purchased from Cell Signaling Technology (Danvers, MA, USA) and GE Healthcare (Chicago, Illinois, USA), respectively.

### Analysis of the cancer dependency map (DepMap) dataset

The dataset published in the release of “DepMap Public 22Q2” (https://depmap.org/portal/download/) was used to assess the impact of *Ppp6c* knockout by genome editing on proliferation of cell lines in the Ppp6c collection [17, 18]. The sgRNA library used in the DepMap project was reported to contain four sgRNA barcodes targeting the *Ppp6c* gene. Cell lines were histologically classified according to the DepMap portal or Expasy operated by the BROAD Institute or the Swiss Institute of Bioinformatics, respectively.

### Statistics

The two‐tailed Student's *t* test was used to determine *P* values. *P* values < 0.01 were considered significant.

## Results

### Establishment of CreERT‐Ppp6c^fl^ ^/^ ^fl^‐K‐Ras^G12V^ MEFs

**Fig. 1:**
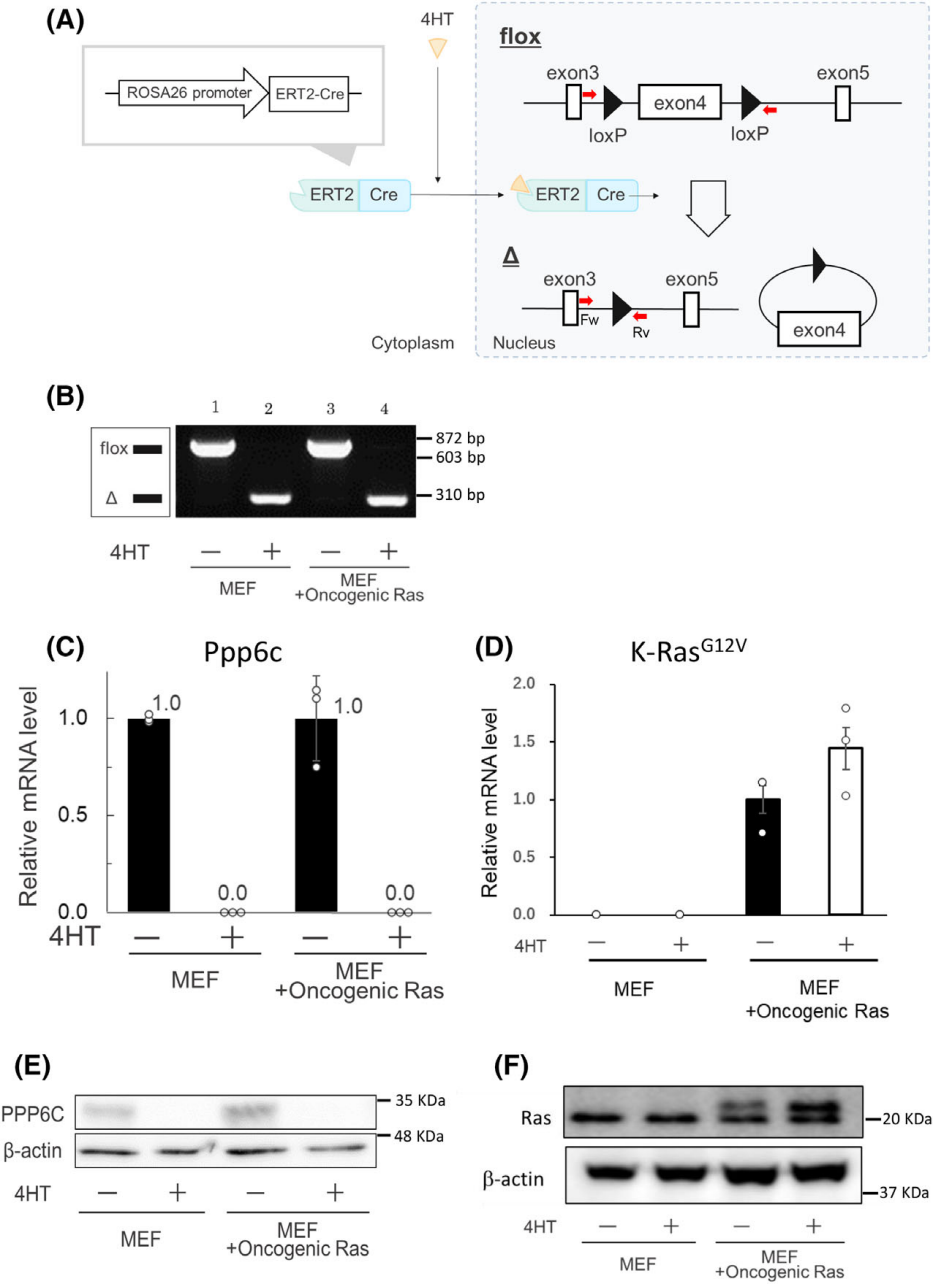
Establishment of CreERT‐Ppp6c^fl/fl^‐K‐Ras^G12V^ MEFs. (A) CreERT‐mediated *Ppp6c* disruption. Schematic representation of the *Ppp6c* floxed allele and deletion of exon 4 by activated CreERT. The positions of the forward and reverse primers are indicated. 4HT, 4‐hydroxytamoxifen; Δ, exon 4‐deleted allele; and flox, floxed allele. (B) PCR analysis of genomic DNA to detect the exon 4‐deleted *Ppp6c* allele using forward and reverse primers. Lanes 1 and 2: genomic DNA from 4HT‐untreated (lane 1) and 4HT‐treated (lane 2) CreERT‐Ppp6c^fl/fl^ MEFs. Lanes 3 and 4: genomic DNA from 4HT‐untreated (lane 3) and 4HT‐treated (lane 4) CreERT‐Ppp6c^fl/fl^‐K‐Ras^G12V^ MEFs. MEF, CreERT‐Ppp6c^fl/fl^ MEFs; MEF + Oncogenic Ras, CreERT‐Ppp6c^fl/fl^‐K‐Ras^G12V^ MEFs; 4HT‐, 4HT‐untreated; and 4HT^+^, 4HT‐treated. (C) *Ppp6c* mRNA expression levels measured by real‐time PCR. Values are expressed relative to mRNA expression of *Ppp6c* in 4HT‐untreated cells. White circles in the graph indicate values for each sample. *n* = 3 for 4HT^+^ and 4HT^−^. MEF, CreERT‐Ppp6c^fl/fl^ MEFs; MEF + Oncogenic Ras, CreERT‐Ppp6c^fl/fl^‐K‐Ras^G12V^ MEFs; 4HT^−^, 4HT‐untreated; and 4HT^+^, 4HT‐treated. Data are presented as mean ± SD of three independent experiments. (D) *K‐Ras* ^*G12V*^ mRNA expression levels measured by real‐time PCR. Values are expressed relative to mRNA expression of *K‐Ras* ^*G12V*^ in 4HT‐untreated CreERT‐Ppp6c^fl/fl^‐K‐Ras^G12V^ MEFs. *n* = 3 for each sample. MEF, CreERT‐Ppp6c^fl/fl^ MEFs; MEF + Oncogenic Ras, CreERT‐Ppp6c^fl/fl^‐K‐Ras^G12V^ MEFs; 4HT^−^, 4HT‐untreated; and 4HT^+^, 4HT‐treated. Data are presented as mean ± SD of three independent experiments. (E) Ppp6c protein expression levels measured by immunoblotting. MEF, CreERT‐Ppp6c^fl/fl^ MEFs; MEF + Oncogenic Ras, CreERT‐Ppp6c^fl/fl^‐K‐Ras^G12V^ MEFs; 4HT^−^, 4HT‐untreated; and 4HT^+^, 4HT‐treated. (F) Ras protein expression levels measured by immunoblotting. The upper band is introduced K‐Ras^G12V^ and the lower band is endogenous Ras [19]. MEF, CreERT‐Ppp6c^fl/fl^ MEFs; MEF + Oncogenic Ras, CreERT‐Ppp6c^fl/fl^‐K‐Ras^G12V^ MEFs; 4HT^−^, 4HT‐untreated; and 4HT^+^, 4HT‐treated.

We previously established CreERT‐Ppp6c^fl/fl^ MEFs [15]. CreERT‐Ppp6c^fl/fl^‐K‐Ras^G12V^ MEFs were produced by transfecting a K‐Ras^G12V^‐expressing retrovirus into CreERT‐Ppp6c^fl/fl^ MEFs. *Ppp6c* deletion was induced by addition of 4HT (Fig. 1A). We confirmed genomic deletion of *Ppp6c* exon 4 (Fig. 1B) and reduced Ppp6c mRNA and protein levels (Fig. 1C,E) in response to addition of 4HT, and mRNA and protein expression of K‐Ras^G12V^ (Fig. 1D,F) [19].

### Loss of Ppp6c greatly reduces proliferation of K‐Ras^G12V^ ‐expressing MEFs

**Fig. 2:**
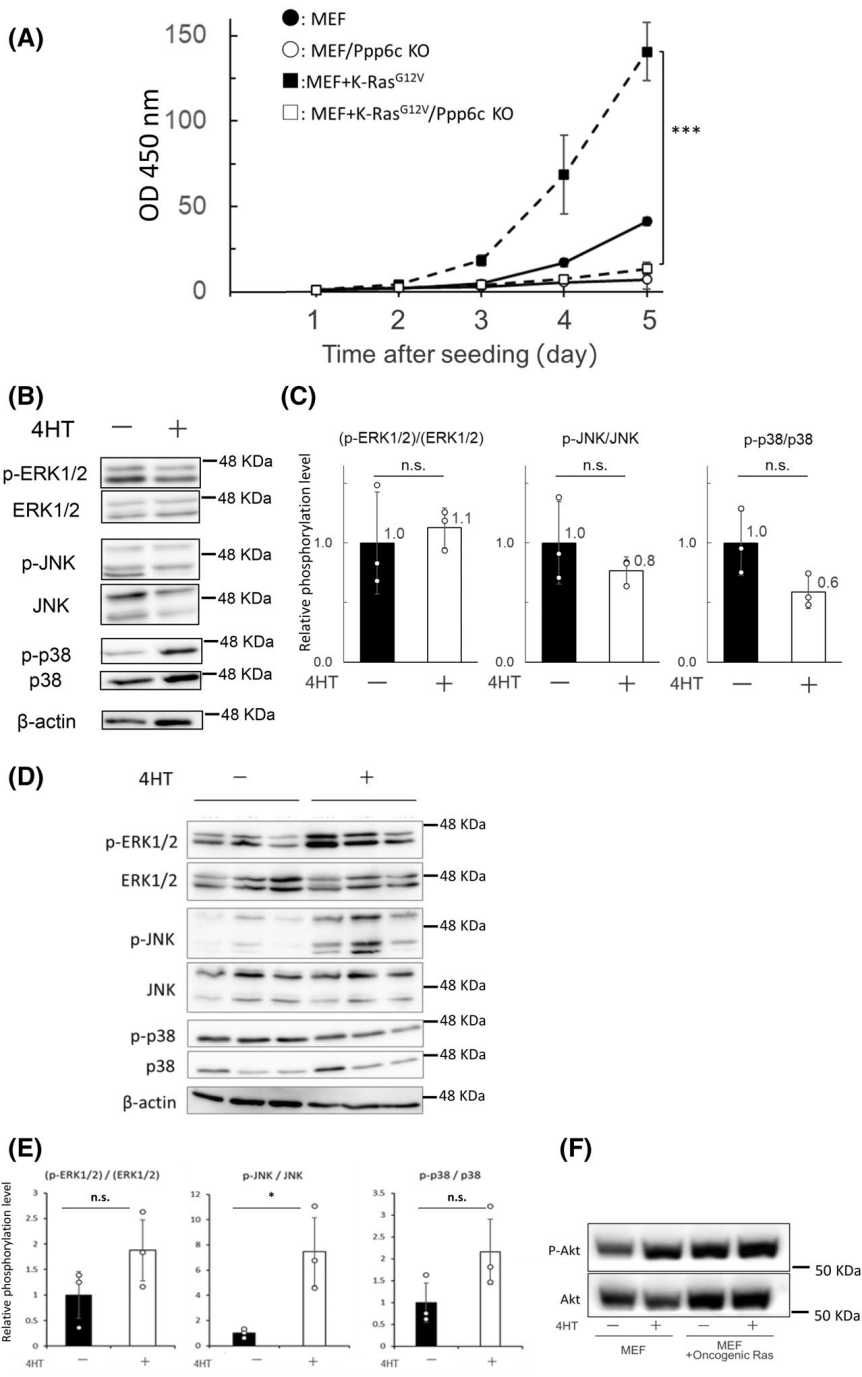
Loss of Ppp6c greatly reduces proliferation of K‐Ras^G12V^‐expressing MEFs and does not markedly activate ERK1/2, JNK, or p38 in these cells. (A) Five hundred cells were seeded per well into a 96‐well plate and cultured for up to 5 days. Cell numbers were determined using a Cell Counting Kit‐8. Values are expressed relative to the OD450 values on Day 1. ●, 4HT‐untreated CreERT‐Ppp6c^fl/fl^ MEFs; ○, 4HT‐treated CreERT‐Ppp6c^fl/fl^ MEFs; ■, 4HT‐untreated CreERT‐Ppp6c^fl/fl^‐K‐Ras^G12V^ MEFs; and □, 4HT‐treated CreERT‐Ppp6c^fl/fl^‐K‐Ras^G12V^ MEFs. For MEFs, *n* = 3 for 4HT^+^ and 4HT^−^. For K‐Ras^G12V^‐expressing MEFs, *n* = 3 for 4HT^+^ and 4HT^−^. Data are presented as mean ± SD of three independent experiments. ***P < 0.001 by the Student's *t* test. (B) Western blotting was performed to examine the effect of Ppp6c deficiency on the phosphorylation levels of MAPKs in K‐Ras^G12V^‐expressing MEFs. 4HT^+^ and 4HT^−^ represent with and without 4HT, respectively. (C) The results from (B) were quantified using imagej. p‐MAPK values were divided by MAPK values and the resulting values were plotted, with the average value in K‐Ras^G12V^‐expressing MEFs without 4HT set to 1. White circles in the graph indicate values for each sample. *n* = 3 for 4HT^+^ and 4HT^−^. Data are presented as mean ± SD of three independent experiments. n.s., *P* > 0.05 by the Student's *t* test. (D) Western blotting was performed to examine the effect of Ppp6c deficiency on the phosphorylation levels of MAPKs in CreERT‐Ppp6c^fl/fl^ MEFs. 4HT^+^ and 4HT^−^ represent with and without 4HT, respectively. (E) The results from (D) were quantified using ImageJ as in (C). White circles in the graph indicate values for each sample. *n* = 3 for 4HT^+^ and 4HT^−^. Data are presented as mean ± SD of three independent experiments. *P < 0.05 by the Student's *t* test. (F) Western blotting was performed to examine the effect of Ppp6c deficiency on the phosphorylation level of Akt in CreERT‐Ppp6c^fl/fl^ MEFs with and without K‐Ras^G12V^. 4HT^+^ and 4HT^−^ represent with and without 4HT, respectively.

First, we analyzed proliferation of CreERT‐Ppp6c^fl/fl^ and CreERT‐Ppp6c^fl/fl^‐K‐Ras^G12V^ MEFs. Both types of MEFs grew well, and CreERT‐Ppp6c^fl/fl^‐K‐Ras^G12V^ MEFs grew more rapidly than CreERT‐Ppp6c^fl/fl^ MEFs (Fig. 2A). This indicated that the introduced K‐Ras^G12V^ was functional in MEFs. In accordance with a previous observation [8], proliferation of 4HT‐treated CreERT‐Ppp6c^fl/fl^ MEFs was markedly decreased. Loss of Ppp6c accelerates growth of oncogenic Ras‐expressing cells in mouse and Drosophila [12, 13, 14]; therefore, we expected it to promote growth of CreERT‐Ppp6c^fl/fl^‐K‐Ras^G12V^ MEFs. To our surprise, proliferation of 4HT‐treated CreERT‐Ppp6c^fl/fl^‐K‐Ras^G12V^ MEFs was greatly decreased.

### Loss of Ppp6c does not markedly activate ERK1/2, JNK, or p38 in K‐Ras^G12V^ ‐expressing MEFs

Loss of Ppp6c in oncogenic K‐Ras‐expressing cells markedly activates ERK1/2 in mice and JNK in *Drosophila* [12, 13, 14], and this is thought to promote cell proliferation. In addition, recent work shows that downregulation of PPP6C in some (but not all) human tumor cell lines activates ERK [20]. Thus, we examined whether Ppp6c deficiency activated ERK1/2, JNK, or p38 MAPK in CreERT‐Ppp6c^fl/fl^‐K‐Ras^G12V^ MEFs. Ppp6c deficiency did not activate ERK1/2, JNK, or p38 MAPK (Fig. 2B,C). We also examined whether Ppp6c deficiency activated ERK1/2, JNK, or p38 MAPK in CreERT‐Ppp6c^fl/fl^ MEFs. Unlike the results for CreERT‐Ppp6c^fl/fl^‐K‐Ras^G12V^ MEFs, Ppp6c deficiency did not activate ERK1/2 or p38 MAPK, but slightly activated JNK (Fig. 2D,E). In addition, we examined the effects of Ppp6c deficiency on activation of Akt in CreERT‐Ppp6c^fl/fl^ and CreERT‐Ppp6c^fl/fl^‐K‐Ras^G12V^ MEFs. Although loss of Ppp6c in oncogenic K‐Ras‐expressing cells activates Akt in mice [13, 14] and in CreERT‐Ppp6c^fl/fl^ MEFs, Ppp6c deficiency did not activate Akt in CreERT‐Ppp6c^fl/fl^‐K‐Ras^G12V^ MEFs (Fig. 2F). These data suggest that the molecular mechanism by which Ppp6c affects proliferation of oncogenic K‐Ras (K‐Ras^G12V^)‐expressing MEFs differs from those reported previously [12, 13, 14].

### Loss of Ppp6c greatly reduces anchorage‐independent growth of K‐Ras^G12V^ ‐expressing MEFs

**Fig. 3:**
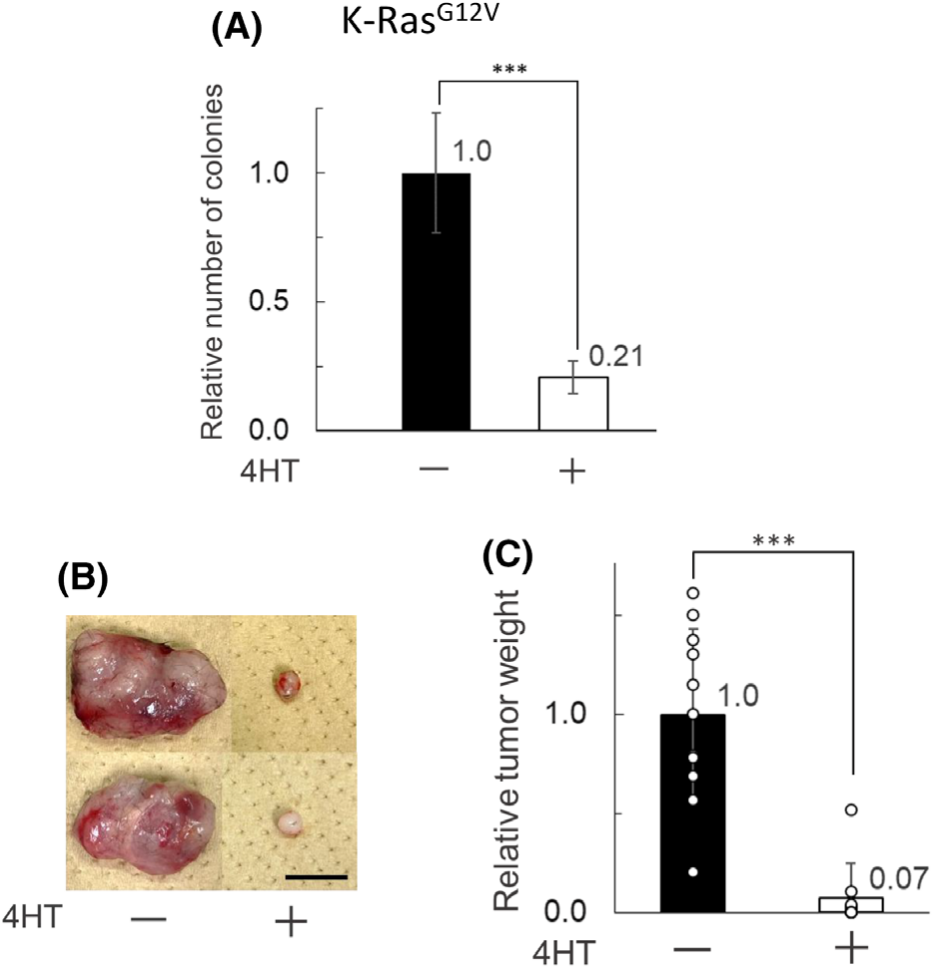
Loss of Ppp6c greatly reduces anchorage‐independent growth of K‐Ras^G12V^‐expressing MEFs and their tumor formation in C57BL/6 mice. (A) Anchorage‐independent growth in 3D culture. 4HT‐untreated or ‐treated CreERT‐Ppp6c^fl/fl^‐K‐Ras^G12V^ MEFs (1.0 × 10^4^) in a 6‐well plate or 3.5 cm dishes were cultured for 20 days in soft agar and stained with Crystal Violet solution. Digital images of the colonies were acquired. Colonies were manually counted. Values are expressed relative to the number of colonies of 4HT‐untreated cells. *N* = 32 for 4HT^+^ and 4HT^−^. Data are presented as mean ± SD of three independent experiments. ***P < 0.001 by the Student's *t* test. 4HT^−^, 4HT‐untreated, and 4HT^+^, 4HT‐treated. (B) Tumors that formed after transplantation of 4HT‐untreated or ‐treated CreERT‐Ppp6c^fl/fl^‐K‐Ras^G12V^ MEFs were photographed. Two typical examples are shown. 4HT^+^ and 4HT^–^ represent with and without 4HT, respectively. Scale bar is 1 cm. (C) The results from (B) were quantified as weight relative to the mean of the 4HT‐treated group. White circles in the graph indicate values for each sample. *n* = 10 for 4HT^+^ and *n* = 11 for 4HT^−^. Data are presented as mean ± SD of three independent experiments. ***P < 0.001 by the Student's *t* test.

Loss of Ppp6c had similar effects on K‐Ras^G12V^‐expressing and normal MEFs under normal *in vitro* culture conditions. Anchorage‐independent growth is a well‐known characteristic of oncogenic cells. Thus, we analyzed the effect of Ppp6c deletion on anchorage‐independent growth. 4HT treatment markedly decreased anchorage‐independent growth of K‐Ras^G12V^‐expressing MEFs (Fig. 3A). The absolute number of colonies was 455 ± 105.7 for 4HT‐untreated cells and 95 ± 28.9 for 4HT‐treated cells. CreERT‐Ppp6c^fl/fl^ MEFs did not form any colonies in this assay as expected. These data indicate that Ppp6c is indispensable for proper anchorage‐independent growth of oncogenic K‐Ras (K‐Ras^G12V^)‐expressing MEFs.

### Loss of Ppp6c greatly reduces tumor formation of K‐Ras^G12V^ ‐expressing MEFs in C57BL/6 mice

Finally, we analyze tumor formation of K‐Ras^G12V^‐expressing MEFs in C57BL/6 mice. CreERT‐Ppp6c^fl/fl^‐K‐Ras^G12V^ MEFs have the C57BL/6 background; therefore, we transplanted them into female C57BL/6 mice. 4HT treatment markedly decreased tumor formation of K‐Ras^G12V^‐expressing MEFs in C57BL/6 mice (Fig. 3B,C). These data indicate that Ppp6c is indispensable for proper tumor formation of oncogenic K‐Ras (K‐Ras^G12V^)‐expressing MEFs in mice.

## Discussion

Dephosphorylation of Aurora kinase A and analysis of gene deletions in *Drosophila* and mouse suggest that Ppp6c plays an essential role in normal cell proliferation. On the other hand, in *Drosophila* and mice, Ppp6c suppresses the effects of oncogenic Ras (Ras^V12^ in *Drosophila* and K‐Ras^G12D^ in mice), and loss of Ppp6c in concert with oncogenic Ras expression accelerates cancer cell growth [12, 13, 14]. These findings support the idea that Ppp6c is not essential for, but rather suppresses, cancer cell proliferation.

However, the effect of loss of Ppp6c at the cellular level in MEFs with or without oncogenic Ras expression has not been analyzed in detail. In the present study, we generated cultured MEFs expressing oncogenic K‐Ras (K‐Ras^G12V^) and analyzed the effect of Ppp6c deficiency to determine how Ppp6c is involved in oncogenic Ras‐induced cancer cell proliferation.

Although loss of Ppp6c was expected to promote proliferation of K‐Ras^G12V^‐expressing MEFs, it markedly inhibited proliferation of these cells under normal culture conditions, their colony formation on soft agar medium, and their tumor formation upon subcutaneous implantation into C57BL/6 mice. In addition, we examined whether Ppp6c deficiency activated the JNK and ERK MAPKs and Akt as reported previously, but such activation was not observed. Contrary to our expectation, Ppp6c appears to be essential for proper proliferation of immortalized MEFs expressing K‐Ras^G12V^. Collectively, these findings show that oncogenic K‐Ras^G12V^ cannot overcome proliferation failure caused by loss of Ppp6c in MEFs.

**Fig. 4:**
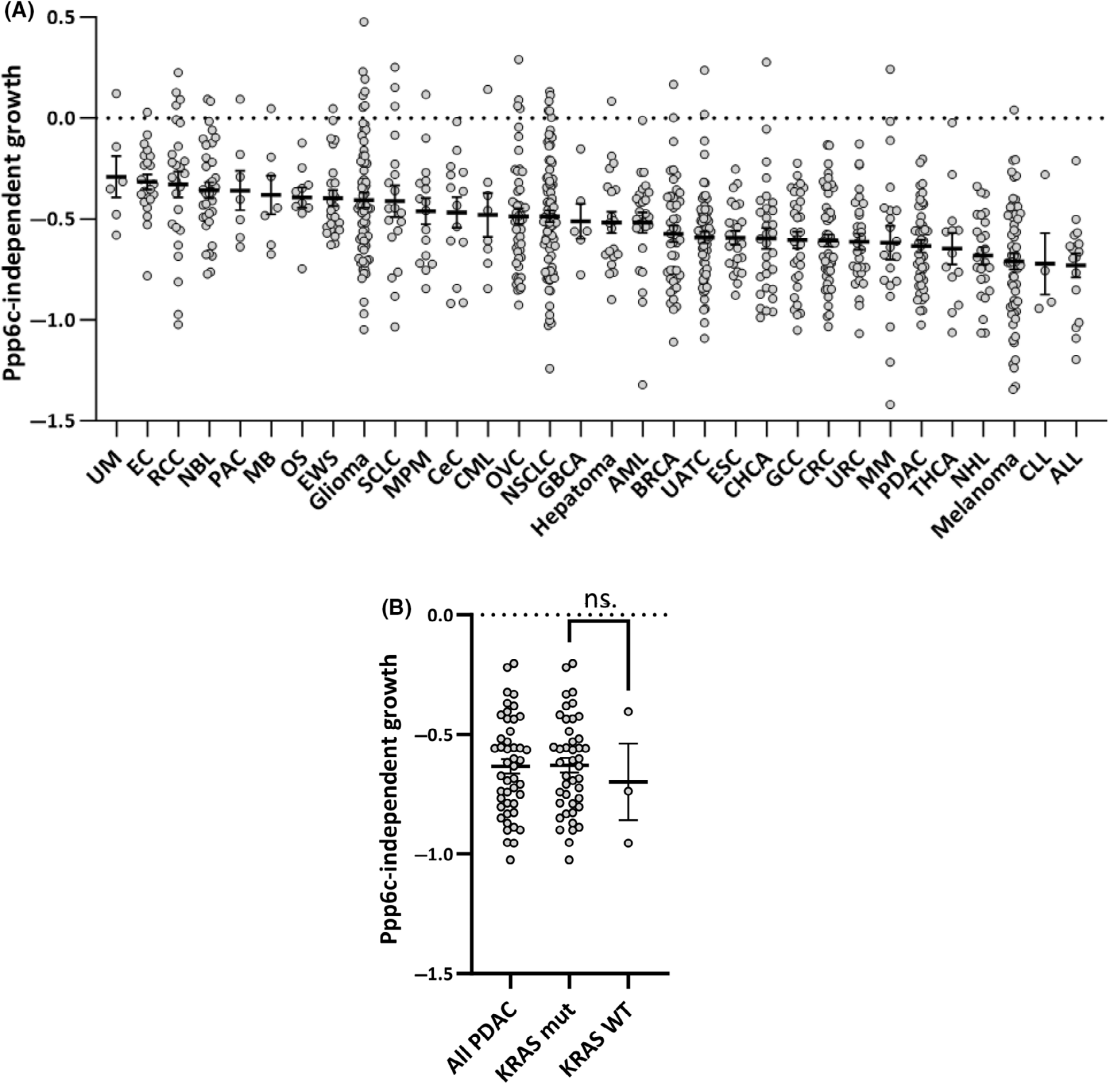
Analysis of the DepMap dataset showing the effects of *PPP6C* knockout on proliferation of cell lines in the CCLE collection. (A) *PPP6C*‐independent growth (Y axis) represents enrichment scores for *PPP6C* sgRNA barcodes in CRISPR/Cas9 loss‐of‐function proliferation screens collected in DepMap. Each symbol shows an individual cell line. Results are summarized for each cancer type and sorted according to the mean *PPP6C* knockout effect in each group. Note that a value less than 0 on the Y axis indicates that cell growth was suppressed upon *PPP6C* knockout. ALL, acute lymphocytic leukemia; AML, acute myeloid leukemia; BRCA, breast cancer; CeC, cervical cancer; CHCA, cholangiocarcinoma; CLL, chronic lymphocytic leukemia; CML, chronic myeloid leukemia; CRC, colorectal cancer; EC, endometrial cancer; ESC, esophagus cancer; EWS, Ewing sarcoma; GBCA, gallbladder adenocarcinoma; GCC, gastric cancer; MB, medulloblastoma; MM, multiple myeloma; MPM, malignant pleural mesothelioma; NBL, neuroblastoma; NHL, non‐Hodgkin lymphoma; NSCLC, non‐small‐cell lung cancer; OS, osteosarcoma; OVC, ovarian cancer; PAC, prostate adenocarcinoma; PDAC, pancreatic ductal adenocarcinoma; RCC, renal cancer; SCLC, small‐cell lung cancer; THCA, thyroid carcinoma; UATC, upper aerodigestive tract cancer; UM, uveal melanoma; URC, urinary bladder carcinoma. (B) The results of 46 PDAC lines in A were further compared after sub‐grouping based on the presence or absence of K‐Ras mutations. Data are presented as mean + SEM. ns, not significant.

Why do our results differ from those reported previously [12, 13, 14]? One possible explanation is that the requirement for Ppp6c for proper proliferation differs between cell types. Recently, Zou *et al*. [21] reported that deletion of Ppp6c does not significantly affect proliferation of L929 cells. We also found that Ppp6c deletion using Nestin‐Cre has little effect on cell proliferation in the brain (manuscript in preparation). Thus, some cell types do not display Ppp6c‐dependent proliferation. It is possible that MEFs are highly dependent on Ppp6c and cannot proliferate upon expression of K‐Ras^G12V^ when Ppp6c is deficient. What about other cancer cell lines? We checked the DepMap database (https://depmap.org/portal/) [17, 18, 22]. In DepMap, the effects of gene knockout using genome editing on proliferation were comprehensively examined in about 1000 human cell lines. We extracted the results for *PPP6C* (Fig. 4A), which showed that most cancer cells require *PPP6C* for proper proliferation. We selected pancreatic invasive ductal adenocarcinoma (PDAC) cell lines and checked K‐Ras mutations. Three of the 46 cell lines had no K‐Ras mutations, and the effect of Ppp6c deficiency did not significantly differ between cell lines with and without K‐Ras mutations (Fig. 4B). Collectively, these findings show that Ppp6c is indispensable for proper proliferation even of cancer cells.

Another possibility is that different types of K‐Ras mutations have different functions. There are several subtypes of oncogenic K‐Ras mutations at G12 such as K‐Ras^G12V^, K‐Ras^G12D^, K‐Ras^G12R^, and K‐Ras^G12C^ [23]. A previous study used K‐Ras^G12D^ in mouse [13, 14], but we used K‐Ras^G12V^. Although the mean Z scores of K‐Ras^G12V^ (−0.65, 14 cases) and K‐Ras^G12D^ (−0.6, 19 cases) are almost identical, these two mutations may have different functions or the effect of loss of Ppp6c on cell proliferation may differ between cells with these two mutations.

Another possibility is that the results differ because the cells were placed in different conditions. For example, normal cells kill cancer cells with which they are in contact, a phenomenon called cell competition [24]. Recent studies established that cell competition functions both as a tumor‐suppressing mechanism and a tumor‐promoting mechanism, and thereby critically influences cancer initiation and development [25]. In our study, oncogenic K‐Ras‐expressing and Ppp6c‐deficient cells were surrounded by normal wild‐type cells; therefore, cell competition activity of surrounding normal cells may have prevented tumor growth. On the other hand, in a previous study using mice, Ppp6c deficiency was also induced in cells surrounding tumor‐forming cells, suggesting that Ppp6c deficiency suppresses cell competition, which increases growth of oncogenic K‐Ras‐expressing and Ppp6c‐deficient cells. To explore this possibility in our MEFs, it might be possible to transplant cells without Ppp6c deficiency and induce this deficiency using 4HT when they have proliferated slightly. However, there are uncertainties, including whether Ppp6c deficiency can be achieved with sufficient efficiency. It may be necessary to create a new cell system for this experiment. This issue should be addressed in the future.

## Conflict of interest

The authors have no financial conflicts of interest.

### Peer review

The peer review history for this article is available at https://www.webofscience.com/api/gateway/wos/peer‐review/10.1002/2211‐5463.13775.

## Author contributions

MI, AK, KM, MS, and SM performed the experiments, analyzed the data, and wrote part of the manuscript; NT, YK, KM, AK, MS, HS, SM, and TW helped to perform the experiments; and MI, NT, SM, and TW designed the experiments and wrote the manuscript.

## Data Availability

The data that support the findings of this study are available from the corresponding author [toshiwatana@cc.nara-wu.ac.jp] upon reasonable request.
